# Supplementary material for: Disparate expression specificities coded by a shared Hox-C enhancer
Source: eLife. 2020 Apr 28;9:e39876. doi: 10.7554/eLife.39876 (PMC7188484; doi:10.7554/eLife.39876)
Supplement: Supplementary file 1. — Phylogenetic compilation of motifs similar to the conserved EO053 Hox-like binding motif in the pb region. Alignments correspond to each of the numbered motifs in Figure 5—figure supplement 3–9; shown is a 24-nt segment including each motif (core 12 nt flanked by 6 nt on each side). [file elife-39876-supp1.docx]

ATCATTAATCAT motifs exhibiting clade-specific patterns of conservation.

Motif numbers refer to Supplemental Figure 4.

**1. Brachycera: Intronic EO053 match:**

Species Strand L flank Motif R flank

Dm - GTTTGC ATCATTAATCAT GACGTT

Da - GTTTGC ATCATTAATCAT GACGTT

Dp - GTTTGC ATCATTAATCAT GACGTT

Dv + GCCACC ATCATTAATCAT GACGTT

Dg + GCCACC ATCATTAATCAT GACGTT

Lc - GTTTGC ATCATTAATCAT GACGTT

Bd - GTTAAC ATCATTAATCAT GACATT

Cc - GTTAAC ATCATTAATCAT GACATT

**2. Lepidoptera: *pb* Upstream match:**

Species Strand L flank Motif R flank

Ll - TTATTG ATCATTAATCAT TATAAT

At + TTTACA ATCATTAATCAT TATTTC

Ms + TTTGCC ATCATTAATCAT TATTTC

Bm + TTTTCT ATCATTAATCAT TATTTC

Dp + TTTGCG ATCATTAATCAT TATTTC

Hv + TTTGCC ATCATTAATCAT TATTTC

**3. Coleoptera: *pb* Upstream mismatch**

Species Strand L flank Motif R flank

Nv + TTAGGC ATCATTAATCAA GTGTTG

Ap + TTAGGC ATCATTAATCAA AAGTTA

Tc - TTAGAC ATCATTAATCAA GCGTTG

Ld - TTAGGC ATCATTAATCAA GTCGGT

Ag - TTAGGC ATCATTAATCAA GTTGGT

Dp + TTAGGC ATCATTAATCAA GATGCT

**4. Coleoptera: *pb* Intron mismatch**

Species Strand L flank Motif R flank

Tc + AATTTT ACTATTAATCAT TTCTTA

Ld + AGTTTT ACTATTAATCAT CTTTAC

Ag + AGTTTT ACTATTAATCAT CTTTAC

**5. Coleoptera: *zen* Upstream mismatch**

Species Strand L flank Motif R flank

Ap - TCCTCG ATCATCAATCAT TCTGAG

Tc - TCCTAG ATCATCAATCAT CCCGAG

At - AATTAA ATCATCAATCAT CATTTT

Ag + TCCTAG ATCATCAATCAT CCCGAG

Ag* - GGAATC ATCATCAATCAT CATTTG

*on separate *zen* duplication scaffold

**6. Hymenoptera: Tenthredinoidea *pb* Downstream mismatch**

Species Strand L flank Motif R flank

Nl - TCCCCA AACATTAATCAT TATCCG

Ar - TCCCCA AACATTAATCAT TATCCG

Cc - TCCCCA GGCATTAATCAT TATCCG

Oa - CCCCAA AGCATTAATCAT TATCCG

**7. Hymenoptera: Tenthredinoidea *pb* Intron mismatch**

Species Strand L flank Motif R flank

Nl - CAAAGA ATCATTAGTCAT TTCTGC

Ar - CAAAGA ATCATTAGTCAT TTCTGT

**8. Hymenoptera: Tenthredinoidea *pb* Upstream mismatch1**

Species Strand L flank Motif R flank

Nl - CGGTGA GATATTAATCAT TTTCCA

Ar - CCCGGA GAGATTAATCAT TTTCAT

**9. Hymenoptera: Tenthredinoidea *pb* Upstream mismatch2**

Species Strand L flank Motif R flank

Nl - CGGGCG TTAATTAATCAT ATATTA

Ar - CTGGTA TTAATTAATCAT ATATAC

**10. Hymenoptera: Tenthredinoidea *zen* Upstream mismatch**

Species Strand L flank Motif R flank

Nl - CCGTCG ATCATTAATCAC GTTCCA

Ar - CCAGCG ATCATTAATCAC GTACAC

**11. Hymenoptera: *zen* Upstream mismatch (absent Tenthredinoidea):**

Species Strand L flank Motif R flank

Cc - CAAACT ATCATCAATCAT CAAGGG

Oa - AGAACG ATCATCAATCAT CAAGAG

Md - CAAACG ATCATCAATCAT CAAGGG

Da - CAAACG ATCATCAATCAT CAAGAC

Fa - CAAACG ATCATCAATCAT CAAGAG

Tp - CAAGGT ATCATCAATCAT TAAGAG

Cf - AAAACC ATCATCAATCAT CGAGAC

Nv - CAAACT ATCATCAATCAT CAAGAA

Hs - CGAACG ATCATCAATCAT CAAGAG

Lh - CGAACG ATCATCAATCAT CAAGAG

Pb - CGAACG ATCATCAATCAT CAAGAG

Si - CGAACG ATCATCAATCAT CAAGAG

Co - CGAACG ATCATCAATCAT CAAGAG

Ac - CGAACG ATCATCAATCAT CAAGAG

Dn - CGAACG ATCATCAATCAT CAAGAG

La - CGAACG ATCATCAATCAT CAAGAG

Ed - CGAACG ATCATCAATCAT CAAGAG

Bi* - CGAACG ATCATCAATCAT CAAGAG

Hl - CGAACG ATCATCAATCAT CAAGAG

Am - GAAACG ATCATCAATCAT CAAGAG

Mq - CAAACG ATCATCAATCAT CAAGAG

*on Dfd scaffold

**12. Hymenoptera: Aculeata *pb* Intron mismatch:**

Species Strand L flank Motif R flank

Hs - TAAATG ATCATTAATTAG GACACC

Lh - TAAATG ATCATTAATTAG AACACC

Pb - TAAATG ATCATTAATTAG GATATC

Si - TAAATG ATCATTAATTAG GATACC

Co - TAAATG ATCATTAATTAG GATACC

Ac - TAAATG ATCATTAATTAG GATACC

Dn - TAAATG ATCATTAATTAA GCGACT

La - TAAATG ATCATTAATTAA ACGACT

Ed - TAAATC ATCATTAATTAA GCGGCC

Bi - TAAATA ATCATTAATTAA GCTGCC

Hl - TAAATG ATCATTAATTAG GAAGCT

Am - TCAACG ATCATTAATTAA GCGGGC

Mq - TAAATG ATCATTAATTAA GCCGCC

**13. Hymenoptera: Formicoidea *pb* Intron mismatch:**

Species Strand L flank Motif R flank

Hs - AGAGAA ATCATTAATCAA GATCGT

Lh - TGGAAC ATCATTAATCAA AGATCG

Pb - AGAGAT ATCATTAATCAA AGATTA

Si - AAAGAT ATCATTAATCAA AGATCG

Co - AGAGAT GCCATTAATCAA AGATCG

Ac - AGAAAT ATCATTAATCAA AGATCA

**14. Hemiptera: *zen* Downstream mismatch (absent in Sternorrhyncha)**

Species Strand L flank Motif R flank

Hv + GCCTTC ATCATTACTCAT TTACCA

Hh + TGCCTC ATCATTACTCAT TTACCC

Of - GAGCTC ATCATTACTCAT TTAACC

Cl + CTCTTC ATCATTACTCAT TTAGCC

Rp + TCCTTC ATCATTACTCAT TTACCT

**15. Dictyoptera: *zen* Upstream mismatch**

Species Strand L flank Motif R flank

Bg - CTTAAG ATCATCAATCAT TTCGCC

Zn - CTTAAG ATCATCAATCAT TTCGCC

**16. Chelicerata: *Hox3/pb* Downstream mismatch**

Species Strand L flank Motif R flank

LpHox3-1 - GAGCAC ATCATAAATCAT ATCTTC

LpHox3-2 - AAGAAC ATCATAAATCAT GTCTGC

LpHox3-3 - AGGCAC ATCATAAATCAT TTCTGA

CeHox3 - CCCATC ATCATAAATCAT CACCGT

SmHox3 - TCTGTG ATCATAAATCAT TCCAAC

PtHox3 - ACTTTC ATCATAAATCAT TCCTCC

LpPb-1 - TCTCCT ATCATTAAGCAT GATAAT

LpPb-2 - TAATAT ATCATAAATCAT GCGCTT

CePb - ACAAGA ATCATAAATCAT CATCCT

PtPb-2 - ATTCAA ATCATAAATCAT GGAATT

PtPb-1 - ACCAAC ATCATTTATCAT ATCTTA

**17. Chelicerata: *Hox3/pb* Intron mismatch**

Species Strand L flank Motif R flank

LpPb-1 - CACAAA TTCATTAATCAT ATGAAC

LpHox3-1 - AAAAAT TTCATTAATCAT AATTTC

LpHox3-3 - TCAAGT TTCATTAATCAT CATTTT

SmPb - AAAGTT TTCATTAATCAT TATTAG
