# Supplementary material for: Disparate expression specificities coded by a shared Hox-C enhancer
Source: eLife. 2020 Apr 28;9:e39876. doi: 10.7554/eLife.39876 (PMC7188484; doi:10.7554/eLife.39876)
Supplement: Supplementary file 3. — Shown are the D. melanogaster and D. virilis endogenous promoters for pb and zen2, noting the presence and location of various promoter elements, as well as the DSCP sequence from the pBPGUw reporter vector (Pfeiffer et al., 2008). Secondly are shown the sequences replacing the DSCP promoter for testing promoter influence on enhancer expression: pb, zen2-modified DSCP, and bcd. [file elife-39876-supp3.docx]

**KEY:**

TATA DPE INR

MTE Dmv3 (Fitzgerald 2006) INR replacement from zen2

**Annotated TSS**

Mismatched consensus nucleotide

***zen2* promoter**

*D.melanogaster*

TCCGATCCGTCACGAAGGGCTTCTGGGGTATATAAGCAAATAAGGAGAATCTAA**A**GGTATCTAGTTAAAAAACATCGGCCACAGCGGTCAGAAGCCAACAAATCGCGCTCTTCAAGAAAAATGTTTGCCATTCA

*D.virilis*

CGATTCGCGACTATATTTACCTGCTGGGTATAAAAGTAAACCAGCGGCATGGAATTGCATCTAGTTAAAAAACAAATGTGTCACAGAACAGAAGTTCAAATAACTCTCGGCAATATGTACAACAATATGAATAC

***pb* promoter**

*D.melanogaster*

TGGATGCGGTGGCTGTCGCTTAGGCCACTCCTTGATACTGGGTCCCAAAATCCCAGTTCAGTGCGTTC**G**GTGCTATTGGATGGAATGGTTGTTGGCTGCTATCTAGCTCTCGGGAACCGCACGAAGCTCCCCAA

*D.virilis (DPEmm)*

TATAGTCGTACAAAATGCTCGTTCTTGGCCACTCCTTGCGACAAGTGCGGACAAGTTTCAGTGCGTTCGGTGCTATTGGATGAAATGGTTGGTTAAGAATCGTGCCCGCACGAAGCAGCGTCGTATTGCCCAAGCCGGAGGG

**Full synthetic core promoter (DSCP) sequence, FseI site to KpnI site (sequence from pBPGUw)**

GGCCGGCC GAGCTCGCCCGGGGATCGAGCGCAGCGGTATAAAAGGGCGCGGGGTGGCTGAGAGCATCAGTTGTGAATGAATGTTCGAGCCGAGCAGACGTGCCGCTGCCTTCGTTAATATCCTTTGAATAAGCCAACTTTGAATCACAAGACGCATACCAAAC

GGTACC

**Sequences replacing DSCP in pBPGUw (between FseI and KpnI sites)**

**Full *pb* sequence, for in between FseI and KpnI**

GGCCGGCC TGGATGCGGTGGCTGTCGCTTAGGCCACTCCTTGATACTGGGTCCCAAAATCCCAGTTCAGTGCGTTC**G**GTGCTATTGGATGGAATGGTTGTTGGCTGCTATCTAGCTCTCGGGAACCGCACGAAGCTCCCCAAAAGCCCTCTGTCTCGCTCTCG

GGTACC

***zen2*-like DSCP**

GGCCGGCC GAGCTCGCCCGGGGATCGAGCGCAGCGGTATAAAAGGGCGCGGGGTGGCTGAGAGCATATCTAGTGAATGAATGTTCGAGCCGAGCAGACGTGCCGCTGCCTTCGTTAATATCCTTTGAATAAGCCAACTTTGAATCACAAGACGCATACCAAAC

GGTACC

***bcd* promoter**

GGCCGGCC TTTTTAAATTACTCAAAAGAATGAACATCGAGGGAGGGCCGCCAATTGTGCCATCTCTAC**A**TCTCTTCGCTCATCCCTAAATAACGGCACTCTGCAGATGCGAAGCAGTGGATCGCAAAAACGCAAAATGTGGGCGAAATAAGTTCGCGAGCGTC

GGTACC
