# Supplementary material for: Disparate expression specificities coded by a shared Hox-C enhancer
Source: eLife. 2020 Apr 28;9:e39876. doi: 10.7554/eLife.39876 (PMC7188484; doi:10.7554/eLife.39876)
Supplement: Supplementary file 4. — All PCR-amplified sequences shown were cloned into pCR8/GW/TOPO. Sequence-verified clones with the same orientation as EO053wt were integrated into pBPGUw by Gateway reaction using LR Clonase II (Pfeiffer et al., 2008). Also included are the sequences at the end of each breakpoint of the pbM2:20 deletion and the HDR template sequence used to generate the deletion of region 2.1. [file elife-39876-supp4.docx]

**Reporter sequences and deletion boundaries**

PCR fragments all TA-cloned into pCR8/GW/TOPO, then integrated into pBPGUw by Gateway LR clonase II reaction. Orientation reflected (promoter 3’ to sequence).

>EO053_WT

CCGGAGCGGCACAATTAGTCTTGCCCATAACAGCAGCTAATAGTTTCGTA

AATAGTTTTGGCCAATATTTACATGAGTTGCCTTCTGCCTGGGCTGCTTG

TGGGTTTTGTTCCTTTTGTCTTTTGCTGCCATTGTGAAAATATTTGTCTT

CAAACGACCCATTGTCCCTGCCCGCTCTCCTGTCATTCGGCACGCATCGG

CTACACTCGGAAATATTTACCCCAATTGTATTTCAACTTTCTTTTCACTT

ATAAAATATTTCTGCAAAATATTCTGAGTTTTAAGTTTGTTAAATAGATT

AAAATGACTTGGTTTTGGAAATAAATATTGTAGAACGCGTTCAGATAAAA

GCCGTCCTTTTCAGGTAAGGTAGTAACCAAGAAGTGGCAAGGTAAATACA

ATATTCTGCTACATACTGAAATTTTTTCGACAACAATTTATGTATCTAAA

TAAAAAATATCTCTGAGTGCACACGCATCCGCAGTCGTCGCCGTTCACTG

CTGACGTGCGTCTGTCTCAAGCTCGTCGTCTCCCATCCGCTTTCTGGCCA

TATCTTTTAGTCTGCGTCTGCGTTTGTATCTAAATCTATGGGTTGTTTCT

GCATCAGTATCTGTATCTGTGTCTCCGCGTTTTTCTCGCCGTTGTCTGCC

TTACAAAACGTGTTCATTACAATCCGCTTGCCATTGCTTCTGCTGCAGCC

GCTGCCGCTGCCGCTGCCGCCGATCGGCCAAATGTGTCAGAGCTGAAACG

TCATGATTAATGATGCAAACATGTGGGCCCGTCTCCGTCTGCCTCTCTGT

GTCCGACCGACTCCGACTACGATTCCCAATCCGACTGCGAGTCCGGCTAC

CTTTACGACTCGAATGCGTTCAGGGGGCAGAGACATAAACAGCCCGGGGA

CAGGGCACGGCATGGGAATGGGAATGGGAATGGAATTGGGAATGCGAACG

GTAACGTTTTATCAAGGTAAATTAGAAGACGAGACACCCAAAATGTAAAC

ATCGAACTGGGTAGCTCTTGGACATTAGGCGAAATTAGAAATTAATCCTG

CCACTAAACCACAGATGGAAACACTTCCAAGGGGGATGGTGTGGGAAGAT

TCGACTCCGATGGTCTGAGCAGGGGTAGAGTATCAATTTTTTGTCTACTT

CTTCTTTTGGGGGGCGGAACTTTAGAGGTCAGGCAAAATTAATGGGAAAT

CCTCATGACTGACAAGTCGGAGTAATAGCATATACACACATCATAGTTGA

TCTATCGAGTGAAGCTCATCGAGGAAGTTCCAAATTATATTTCGAGAAAA

AAGGCCTTATATTGGGGAATTGATAAAATAGATCATATATTTATAACCCT

TATCTAAGAAGGCTACGTATTTGAAAGGTTCATTCAGCATTACCG

>DvEO053

TTTGGCTATCAGTAATCGTACTGGTAAAATTAAAGTACAAATATATTTGA

GTTTAATACAAATTAAAACCAATTTGGCCAGCTTTTTGTAGAGTGGAACA

GAAACTTTCCACCTATTTTTTTCAAATGCCCTGGTTCTTTGGCTAACACG

TTTTTGAGCACTAATCCCAGCAAAAGCCTGGGTTTCCTTAAAGGGTATTC

CATCAAGTGCCAAATAAATTCTAATAGTACTAAATATATCGTTCAGTCTT

TAACGCTACTAAGCTCTTCACACACCGAAGATAACAGCTTGTTCTTGTTT

ATTATAGTTAAATTTAGCTGTGCTTGATTTATAGTAGTATTAATCACATA

TTTGTGAATGTATGTTGTATCTATTCCACATATTCATCCCCCAAATGTGA

ATGTTTGTCTTATGACATTGTGTTCCCGCCCCTTGAGCAGTTGTTGCCAT

GGTCAAGACACAACAATACCAATGAGACGCCGCGAGTGCAGCCCAGGATT

AATTTCTAATTTCGCCTAATGCTCCGCCAAGTGTTTCTGTTTGTGTTTGG

CGCGATGTTTACATTTTGTTTGTCGTGTTTTTTTTTTGTTTTTGTCTAAT

TTACCTTGATAAAATGATGCCAGAGCCAGTGCCACCGCCAGTTGTGGTCC

CACTTCCATTCCCAAACCCATTCTCGATGTTTGTGTGTCCCTTCCCATGG

CCCAGTTGCCAGGTCGCACTGCCCTACCCCCAGGCTGTCTCCGTCCCGGC

CTCTAAAAACAGCCCAGTCCGGACTCTTTGGCTCGGGCTAGGGCTCGGTC

TGTGGCTCTGGCTTGGCCTTCGGGCCCACATGCTTGCCACCATCATTAAT

CATGACGTTTCAGCTCTGACACATTTGCCCGATCGGCAGCAGCAGCAGCG

GCGGCCGCGGCCACTGCGGCGGCGGCTACGCACTATTGTAATGAAAACGT

TTTTAAAGCCCTCAGACTGACGGCTGCCTCGTCGAGACGAAGAGACGACC

CGGCCGAGTGTCGGGCGCACAGCGGGCCCCTGGAAGACGGCAAGGCGATG

GACAATGAAATCGAGAGATGAGCAATTTGAAGGCAAATATTTTCATAATG

CGACCACAACACAACCCACACGCCGCACACTCACATGTAAATATTGGCCA

ACTATTTACGAACTATTAGCCACAGTTATGGACAGACTAATTGTAGCTGA

GAATTATAGCAGAGGCAGGCGGCTTAGCATGAATGTGATGATAGGTGCAC

ACCTCTTAGAGGAATTGTTACAATTTGCGCGGGAAATGCAAACTTAATGA

GTTACTTTAATGGGCTCATTTAGTAAAGTTTATAGATTCTCATGAGTAGA

ATGCGCAATAAATTGGGGCTGGCCTTATATATTTTATGAGTGAGTTATAA

TTTTTTTTTGGAAATCAGGGTTCGAAGTTTTGAGTTTGAGTTTAATGTGC

AATCAAAATATAGAAAATATATGATTATTTATTTCAACAAAATAAATTAA

ATATAATTTCAAAGTGTTAATTTTTTGTAGACTTGATTAATGTAAAAGTG

GAAGGAATAATTATCAAAACTCAAAAAATACTTGACAAATGTTTGAAACT

TTCAGCCCAAGAACGATTATGTACAAGAAATTCTATAAGAGTATGTGAAA

CCTATAGCTTTTTAAAAATAAGAATATTTTAAAATAATTTTTGAAATCTA

AAGGATTTATATTGCAAGATGTTTTAGGAAGAAAATATATTTTGTATAGA

ACATTGCCGTCATATCTACAATTCCTTTGCCCTGACGACCCGCTAGCCAA

TAAGGTAAAAGCATTCAGGGTTGGGTAAGAGGTCAAAGGGTGGTCTGGCA

AAAGGGCATATGCTGCTGCTGCCGGCTGCTTCACTGTGCAAGCGATACAA

TAATTGCTCTGGGCCATCGTCGCCGTCGTCATTGTCATTGTTGCGGCTTT

GGCATGATATACAAAAAAAATAAAAATAACAACAAAAACTAATACAACAG

AACAAGAAT

>trunc1

CCGGAGCGGCACAATTAGTCTTGCCCATAACAGCAGCTAATAGTTTCGT

AAATAGTTTTGGCCAATATTTACATGAGTTGCCTTCTGCCTGGGCTGCTT

GTGGGTTTTGTTCCTTTTGTCTTTTGCTGCCATTGTGAAAATATTTGTCT

TCAAACGACCCATTGTCCCTGCCCGCTCTCCTGTCATTCGGCACGCATCG

GCTACACTCGGAAATATTTACCCCAATTGTATTTCAACTTTCTTTTCACT

TATAAAATATTTCTGCAAAATATTCTGAGTTTTAAGTTTGTTAAATAGAT

TAAAATGACTTGGTTTTGGAAATAAATATTGTAGAACGCGTTCAGATAAA

AGCCGTCCTTTTCAGGTAAGGTAGTAACCAAGAAGTGGCAAGGTAAATAC

AATATTCTGCTACATACTGAAATTTTTTCGACAACAATTTATGTATCTAA

ATAAAAAATATCTCTGAGTGCACACGCATCCGCAGTCGTCGCCGTTCACT

GCTGACGTGCGTCTGTCTCAAGCTCGTCGTCTCCCATCCGCTTTCTGG

>trunc2

AATACAATATTCTGCTACATACTGAAATTTTTTCGACAACAATTTATGTA

TCTAAATAAAAAATATCTCTGAGTGCACACGCATCCGCAGTCGTCGCCGT

TCACTGCTGACGTGCGTCTGTCTCAAGCTCGTCGTCTCCCATCCGCTTTC

TGGCCATATCTTTTAGTCTGCGTCTGCGTTTGTATCTAAATCTATGGGTT

GTTTCTGCATCAGTATCTGTATCTGTGTCTCCGCGTTTTTCTCGCCGTTG

TCTGCCTTACAAAACGTGTTCATTACAATCCGCTTGCCATTGCTTCTGCT

GCAGCCGCTGCCGCTGCCGCTGCCGCCGATCGGCCAAATGTGTCAGAGCT

GAAACGTCATGATTAATGATGCAAACATGTGGGCCCGTCTCCGTCTGCCT

CTCTGTGTCCGACCGACTCCGACTACGATTCCCAATCCGACTGCGAGTCC

GGCTACCTTTACGACTCGAATGCGTTCAGGGGGCAGAGACATAAACAGCC

CGGGGACAGGGCACGGCATGGGAATGGGAATGGGAATGGAATTGGGAATG

CGAACGGTAACGTTTTATCAAGGTAAATTAGAAGACGAGACACCCAAAAT

GTAAACATCGAACTGGGTAGCTCTTGGAC

>trunc3

CGGCTACCTTTACGACTCGAATGCGTTCAGGGGGCAGAGACATAAACAGC

CCGGGGACAGGGCACGGCATGGGAATGGGAATGGGAATGGAATTGGGAAT

GCGAACGGTAACGTTTTATCAAGGTAAATTAGAAGACGAGACACCCAAAA

TGTAAACATCGAACTGGGTAGCTCTTGGACATTAGGCGAAATTAGAAATT

AATCCTGCCACTAAACCACAGATGGAAACACTTCCAAGGGGGATGGTGTG

GGAAGATTCGACTCCGATGGTCTGAGCAGGGGTAGAGTATCAATTTTTTG

TCTACTTCTTCTTTTGGGGGGCGGAACTTTAGAGGTCAGGCAAAATTAAT

GGGAAATCCTCATGACTGACAAGTCGGAGTAATAGCATATACACACATCA

TAGTTGATCTATCGAGTGAAGCTCATCGAGGAAGTTCCAAATTATATTTC

GAGAAAAAAGGCCTTATATTGGGGAATTGATAAAATAGATCATATATTTA

TAACCCTTATCTAAGAAGGCTACGTATTTGAAAGGTTCATTCAGCATTAC

CG

>truncA

CCGGAGCGGCACAATTAGTCTTGCCCATAACAGCAGCTAATAGTTTCGTA

AATAGTTTTGGCCAATATTTACATGAGTTGCCTTCTGCCTGGGCTGCTTG

TGGGTTTTGTTCCTTTTGTCTTTTGCTGCCATTGTGAAAATATTTGTCTT

CAAACGACCCATTGTCCCTGCCCGCTCTCCTGTCATTCGGCACGCATCGG

CTACACTCGGAAATATTTACCCCAATTGTATTTCAACTTTCTTTTCACTT

>truncB

CTGCCATTGTGAAAATATTTGTCTT

CAAACGACCCATTGTCCCTGCCCGCTCTCCTGTCATTCGGCACGCATCGG

CTACACTCGGAAATATTTACCCCAATTGTATTTCAACTTTCTTTTCACTT

ATAAAATATTTCTGCAAAATATTCTGAGTTTTAAGTTTGTTAAATAGATT

AAAATGACTTGGTTTTGGAAATAAATATTGTAGAACGCGTTCAGATAAAA

GCCGTCCTTTTCAGGTAAGGTAGTA

>truncC

ATAAAATATTTCTGCAAAATATTCTGAGTTTTAAGTTTGTTAAATAGATT

AAAATGACTTGGTTTTGGAAATAAATATTGTAGAACGCGTTCAGATAAAA

GCCGTCCTTTTCAGGTAAGGTAGTAACCAAGAAGTGGCAAGGTAAATACA

ATATTCTGCTACATACTGAAATTTTTTCGACAACAATTTATGTATCTAAA

TAAAAAATATCTCTGAGTGCACACGCATCCGCAGTCGTCGCCGTTCACTG

>truncD

ACCAAGAAGTGGCAAGGTAAATACA

ATATTCTGCTACATACTGAAATTTTTTCGACAACAATTTATGTATCTAAA

TAAAAAATATCTCTGAGTGCACACGCATCCGCAGTCGTCGCCGTTCACTG

CTGACGTGCGTCTGTCTCAAGCTCGTCGTCTCCCATCCGCTTTCTGGCCA

TATCTTTTAGTCTGCGTCTGCGTTTGTATCTAAATCTATGGGTTGTTTCT

GCATCAGTATCTGTATCTGTGTCTC

>truncE
CTGACGTGCGTCTGTCTCAAGCTCGTCGTCTCCCATCCGCTTTCTGGCCA

TATCTTTTAGTCTGCGTCTGCGTTTGTATCTAAATCTATGGGTTGTTTCT

GCATCAGTATCTGTATCTGTGTCTCCGCGTTTTTCTCGCCGTTGTCTGCC

TTACAAAACGTGTTCATTACAATCCGCTTGCCATTGCTTCTGCTGCAGCC

GCTGCCGCTGCCGCTGCCGCCGATCGGCCAAATGTGTCAGAGCTGAAACG

>truncF

CGCGTTTTTCTCGCCGTTGTCTGCC

TTACAAAACGTGTTCATTACAATCCGCTTGCCATTGCTTCTGCTGCAGCC

GCTGCCGCTGCCGCTGCCGCCGATCGGCCAAATGTGTCAGAGCTGAAACG

TCATGATTAATGATGCAAACATGTGGGCCCGTCTCCGTCTGCCTCTCTGT

GTCCGACCGACTCCGACTACGATTCCCAATCCGACTGCGAGTCCGGCTAC

CTTTACGACTCGAATGCGTTCAGGG

>truncG

TCATGATTAATGATGCAAACATGTGGGCCCGTCTCCGTCTGCCTCTCTGT

GTCCGACCGACTCCGACTACGATTCCCAATCCGACTGCGAGTCCGGCTAC

CTTTACGACTCGAATGCGTTCAGGGGGCAGAGACATAAACAGCCCGGGGA

CAGGGCACGGCATGGGAATGGGAATGGGAATGGAATTGGGAATGCGAACG

GTAACGTTTTATCAAGGTAAATTAGAAGACGAGACACCCAAAATGTAAAC

>truncH

GGCAGAGACATAAACAGCCCGGGGA

CAGGGCACGGCATGGGAATGGGAATGGGAATGGAATTGGGAATGCGAACG

GTAACGTTTTATCAAGGTAAATTAGAAGACGAGACACCCAAAATGTAAAC

ATCGAACTGGGTAGCTCTTGGACATTAGGCGAAATTAGAAATTAATCCTG

CCACTAAACCACAGATGGAAACACTTCCAAGGGGGATGGTGTGGGAAGAT

TCGACTCCGATGGTCTGAGCAGGGG

>truncI

ATCGAACTGGGTAGCTCTTGGACATTAGGCGAAATTAGAAATTAATCCTG

CCACTAAACCACAGATGGAAACACTTCCAAGGGGGATGGTGTGGGAAGAT

TCGACTCCGATGGTCTGAGCAGGGGTAGAGTATCAATTTTTTGTCTACTT

CTTCTTTTGGGGGGCGGAACTTTAGAGGTCAGGCAAAATTAATGGGAAAT

CCTCATGACTGACAAGTCGGAGTAATAGCATATACACACATCATAGTTGA

>truncJ

TAGAGTATCAATTTTTTGTCTACTT

CTTCTTTTGGGGGGCGGAACTTTAGAGGTCAGGCAAAATTAATGGGAAAT

CCTCATGACTGACAAGTCGGAGTAATAGCATATACACACATCATAGTTGA

TCTATCGAGTGAAGCTCATCGAGGAAGTTCCAAATTATATTTCGAGAAAA

AAGGCCTTATATTGGGGAATTGATAAAATAGATCATATATTTATAACCCT

TATCTAAGAAGGCTACGTATTTGAAAGGTTCATTCAGCATTACCG

>trunc1-2

CCGGAGCGGCACAATTAGTCTTGCCCATAACAGCAGCTAATAGTTTCGTA

AATAGTTTTGGCCAATATTTACATGAGTTGCCTTCTGCCTGGGCTGCTTG

TGGGTTTTGTTCCTTTTGTCTTTTGCTGCCATTGTGAAAATATTTGTCTT

CAAACGACCCATTGTCCCTGCCCGCTCTCCTGTCATTCGGCACGCATCGG

CTACACTCGGAAATATTTACCCCAATTGTATTTCAACTTTCTTTTCACTT

ATAAAATATTTCTGCAAAATATTCTGAGTTTTAAGTTTGTTAAATAGATT

AAAATGACTTGGTTTTGGAAATAAATATTGTAGAACGCGTTCAGATAAAA

GCCGTCCTTTTCAGGTAAGGTAGTAACCAAGAAGTGGCAAGGTAAATACA

ATATTCTGCTACATACTGAAATTTTTTCGACAACAATTTATGTATCTAAA

TAAAAAATATCTCTGAGTGCACACGCATCCGCAGTCGTCGCCGTTCACTG

CTGACGTGCGTCTGTCTCAAGCTCGTCGTCTCCCATCCGCTTTCTGGCCA

TATCTTTTAGTCTGCGTCTGCGTTTGTATCTAAATCTATGGGTTGTTTCT

GCATCAGTATCTGTATCTGTGTCTCCGCGTTTTTCTCGCCGTTGTCTGCC

TTACAAAACGTGTTCATTACAATCCGCTTGCCATTGCTTCTGCTGCAGCC

GCTGCCGCTGCCGCTGCCGCCGATCGGCCAAATGTGTCAGAGCTGAAACG

TCATGATTAATGATGCAAACATGTGGGCCCGTCTCCGTCTGCCTCTCTGT

GTCCGACCGACTCCGACTACGATTCCCAATCCGACTGCGAGTCCGGCTAC

CTTTACGACTCGAATGCGTTCAGGGGGCAGAGACATAAACAGCCCGGGGA

CAGGGCACGGCATGGGAATGGGAATGGGAATGGAATTGGGAATGCGAACG

GTAACGTTTTATCAAGGTAAATTAGAAGACGAGACACCCAAAATGTAAAC

ATCGAACTGGGTAGCTCTTGGAC

>trunc2-3

AATACAATATTCTGCTACATACTGAAATTTTTTCGACAACAATTTATGTA

TCTAAATAAAAAATATCTCTGAGTGCACACGCATCCGCAGTCGTCGCCGT

TCACTGCTGACGTGCGTCTGTCTCAAGCTCGTCGTCTCCCATCCGCTTTC

TGGCCATATCTTTTAGTCTGCGTCTGCGTTTGTATCTAAATCTATGGGTT

GTTTCTGCATCAGTATCTGTATCTGTGTCTCCGCGTTTTTCTCGCCGTTG

TCTGCCTTACAAAACGTGTTCATTACAATCCGCTTGCCATTGCTTCTGCT

GCAGCCGCTGCCGCTGCCGCTGCCGCCGATCGGCCAAATGTGTCAGAGCT

GAAACGTCATGATTAATGATGCAAACATGTGGGCCCGTCTCCGTCTGCCT

CTCTGTGTCCGACCGACTCCGACTACGATTCCCAATCCGACTGCGAGTCC

GGCTACCTTTACGACTCGAATGCGTTCAGGGGGCAGAGACATAAACAGCC

CGGGGACAGGGCACGGCATGGGAATGGGAATGGGAATGGAATTGGGAATG

CGAACGGTAACGTTTTATCAAGGTAAATTAGAAGACGAGACACCCAAAAT

GTAAACATCGAACTGGGTAGCTCTTGGACATTAGGCGAAATTAGAAATTA

ATCCTGCCACTAAACCACAGATGGAAACACTTCCAAGGGGGATGGTGTGG

GAAGATTCGACTCCGATGGTCTGAGCAGGGGTAGAGTATCAATTTTTTGT

CTACTTCTTCTTTTGGGGGGCGGAACTTTAGAGGTCAGGCAAAATTAATG

GGAAATCCTCATGACTGACAAGTCGGAGTAATAGCATATACACACATCAT

AGTTGATCTATCGAGTGAAGCTCATCGAGGAAGTTCCAAATTATATTTCG

AGAAAAAAGGCCTTATATTGGGGAATTGATAAAATAGATCATATATTTAT

AACCCTTATCTAAGAAGGCTACGTATTTGAAAGGTTCATTCAGCATTACC

G

>truncA-D

CCGGAGCGGCACAATTAGTCTTGCCCATAACAGCAGCTAATAGTTTCGTA

AATAGTTTTGGCCAATATTTACATGAGTTGCCTTCTGCCTGGGCTGCTTG

TGGGTTTTGTTCCTTTTGTCTTTTGCTGCCATTGTGAAAATATTTGTCTT

CAAACGACCCATTGTCCCTGCCCGCTCTCCTGTCATTCGGCACGCATCGG

CTACACTCGGAAATATTTACCCCAATTGTATTTCAACTTTCTTTTCACTT

ATAAAATATTTCTGCAAAATATTCTGAGTTTTAAGTTTGTTAAATAGATT

AAAATGACTTGGTTTTGGAAATAAATATTGTAGAACGCGTTCAGATAAAA

GCCGTCCTTTTCAGGTAAGGTAGTAACCAAGAAGTGGCAAGGTAAATACA

ATATTCTGCTACATACTGAAATTTTTTCGACAACAATTTATGTATCTAAA

TAAAAAATATCTCTGAGTGCACACGCATCCGCAGTCGTCGCCGTTCACTG

CTGACGTGCGTCTGTCTCAAGCTCGTCGTCTCCCATCCGCTTTCTGGCCA

TATCTTTTAGTCTGCGTCTGCGTTTGTATCTAAATCTATGGGTTGTTTCT

GCATCAGTATCTGTATCTGTGTCTC

>truncF-J

CGCGTTTTTCTCGCCGTTGTCTGCCTTACAAAACGTGTTCATTACAATCC

GCTTGCCATTGCTTCTGCTGCAGCCGCTGCCGCTGCCGCTGCCGCCGATC

GGCCAAATGTGTCAGAGCTGAAACGTCATGATTAATGATGCAAACATGTG

GGCCCGTCTCCGTCTGCCTCTCTGTGTCCGACCGACTCCGACTACGATTC

CCAATCCGACTGCGAGTCCGGCTACCTTTACGACTCGAATGCGTTCAGGG

GGCAGAGACATAAACAGCCCGGGGACAGGGCACGGCATGGGAATGGGAAT

GGGAATGGAATTGGGAATGCGAACGGTAACGTTTTATCAAGGTAAATTAG

AAGACGAGACACCCAAAATGTAAACATCGAACTGGGTAGCTCTTGGACAT

TAGGCGAAATTAGAAATTAATCCTGCCACTAAACCACAGATGGAAACACT

TCCAAGGGGGATGGTGTGGGAAGATTCGACTCCGATGGTCTGAGCAGGGG

TAGAGTATCAATTTTTTGTCTACTTCTTCTTTTGGGGGGCGGAACTTTAG

AGGTCAGGCAAAATTAATGGGAAATCCTCATGACTGACAAGTCGGAGTAA

TAGCATATACACACATCATAGTTGATCTATCGAGTGAAGCTCATCGAGGA

AGTTCCAAATTATATTTCGAGAAAAAAGGCCTTATATTGGGGAATTGATA

AAATAGATCATATATTTATAACCCTTATCTAAGAAGGCTACGTATTTGAA

AGGTTCATTCAGCATTACCG

>truncΔFG

CCGGAGCGGCACAATTAGTCTTGCCCATAACAGCAGCTAATAGTTTCGTA

AATAGTTTTGGCCAATATTTACATGAGTTGCCTTCTGCCTGGGCTGCTTG

TGGGTTTTGTTCCTTTTGTCTTTTGCTGCCATTGTGAAAATATTTGTCTT

CAAACGACCCATTGTCCCTGCCCGCTCTCCTGTCATTCGGCACGCATCGG

CTACACTCGGAAATATTTACCCCAATTGTATTTCAACTTTCTTTTCACTT

ATAAAATATTTCTGCAAAATATTCTGAGTTTTAAGTTTGTTAAATAGATT

AAAATGACTTGGTTTTGGAAATAAATATTGTAGAACGCGTTCAGATAAAA

GCCGTCCTTTTCAGGTAAGGTAGTAACCAAGAAGTGGCAAGGTAAATACA

ATATTCTGCTACATACTGAAATTTTTTCGACAACAATTTATGTATCTAAA

TAAAAAATATCTCTGAGTGCACACGCATCCGCAGTCGTCGCCGTTCACTG

CTGACGTGCGTCTGTCTCAAGCTCGTCGTCTCCCATCCGCTTTCTGGCCA

TATCTTTTAGTCTGCGTCTGCGTTTGTATCTAAATCTATGGGTTGTTTCT

GCATCAGTATCTGTATCTGTGTCTC

ATCGAACTGGGTAGCTCTTGGACATTAGGCGAAATTAGAAATTAATCCTG

CCACTAAACCACAGATGGAAACACTTCCAAGGGGGATGGTGTGGGAAGAT

TCGACTCCGATGGTCTGAGCAGGGGTAGAGTATCAATTTTTTGTCTACTT

CTTCTTTTGGGGGGCGGAACTTTAGAGGTCAGGCAAAATTAATGGGAAAT

CCTCATGACTGACAAGTCGGAGTAATAGCATATACACACATCATAGTTGA

TCTATCGAGTGAAGCTCATCGAGGAAGTTCCAAATTATATTTCGAGAAAA

AAGGCCTTATATTGGGGAATTGATAAAATAGATCATATATTTATAACCCT

TATCTAAGAAGGCTACGTATTTGAAAGGTTCATTCAGCATTACCG

>EO053_FG1m

CCGGAGCGGCACAATTAGTCTTGCCCATAACAGCAGCTAATAGTTTCGTA

AATAGTTTTGGCCAATATTTACATGAGTTGCCTTCTGCCTGGGCTGCTTG

TGGGTTTTGTTCCTTTTGTCTTTTGCTGCCATTGTGAAAATATTTGTCTT

CAAACGACCCATTGTCCCTGCCCGCTCTCCTGTCATTCGGCACGCATCGG

CTACACTCGGAAATATTTACCCCAATTGTATTTCAACTTTCTTTTCACTT

ATAAAATATTTCTGCAAAATATTCTGAGTTTTAAGTTTGTTAAATAGATT

AAAATGACTTGGTTTTGGAAATAAATATTGTAGAACGCGTTCAGATAAAA

GCCGTCCTTTTCAGGTAAGGTAGTAACCAAGAAGTGGCAAGGTAAATACA

ATATTCTGCTACATACTGAAATTTTTTCGACAACAATTTATGTATCTAAA

TAAAAAATATCTCTGAGTGCACACGCATCCGCAGTCGTCGCCGTTCACTG

CTGACGTGCGTCTGTCTCAAGCTCGTCGTCTCCCATCCGCTTTCTGGCCA

TATCTTTTAGTCTGCGTCTGCGTTTGTATCTAAATCTATGGGTTGTTTCT

GCATCAGTATCTGTATCTGTGTCTC

ATATGGGGGAGATAATGGTGAGTAAGGCACCCCATGTGGACGGCACCTCC

GCTTGCCATTGCTTCTGCTGCAGCCGCTGCCGCTGCCGCTGCCGCCGATC

GGCCAAATGTGTCAGAGCTGAAACGTCATGATTAATGATGCAAACATGTG

GGCCCGTCTCCGTCTGCCTCTCTGTGTCCGACCGACTCCGACTACGATTC

CCAATCCGACTGCGAGTCCGGCTACCTTTACGACTCGAATGCGTTCAGGG

GGCAGAGACATAAACAGCCCGGGGACAGGGCACGGCATGGGAATGGGAAT

GGGAATGGAATTGGGAATGCGAACGGTAACGTTTTATCAAGGTAAATTAG

AAGACGAGACACCCAAAATGTAAAC

ATCGAACTGGGTAGCTCTTGGACATTAGGCGAAATTAGAAATTAATCCTG

CCACTAAACCACAGATGGAAACACTTCCAAGGGGGATGGTGTGGGAAGAT

TCGACTCCGATGGTCTGAGCAGGGGTAGAGTATCAATTTTTTGTCTACTT

CTTCTTTTGGGGGGCGGAACTTTAGAGGTCAGGCAAAATTAATGGGAAAT

CCTCATGACTGACAAGTCGGAGTAATAGCATATACACACATCATAGTTGA

TCTATCGAGTGAAGCTCATCGAGGAAGTTCCAAATTATATTTCGAGAAAA

AAGGCCTTATATTGGGGAATTGATAAAATAGATCATATATTTATAACCCT

TATCTAAGAAGGCTACGTATTTGAAAGGTTCATTCAGCATTACCG

>EO053_FG2m

CCGGAGCGGCACAATTAGTCTTGCCCATAACAGCAGCTAATAGTTTCGTA

AATAGTTTTGGCCAATATTTACATGAGTTGCCTTCTGCCTGGGCTGCTTG

TGGGTTTTGTTCCTTTTGTCTTTTGCTGCCATTGTGAAAATATTTGTCTT

CAAACGACCCATTGTCCCTGCCCGCTCTCCTGTCATTCGGCACGCATCGG

CTACACTCGGAAATATTTACCCCAATTGTATTTCAACTTTCTTTTCACTT

ATAAAATATTTCTGCAAAATATTCTGAGTTTTAAGTTTGTTAAATAGATT

AAAATGACTTGGTTTTGGAAATAAATATTGTAGAACGCGTTCAGATAAAA

GCCGTCCTTTTCAGGTAAGGTAGTAACCAAGAAGTGGCAAGGTAAATACA

ATATTCTGCTACATACTGAAATTTTTTCGACAACAATTTATGTATCTAAA

TAAAAAATATCTCTGAGTGCACACGCATCCGCAGTCGTCGCCGTTCACTG

CTGACGTGCGTCTGTCTCAAGCTCGTCGTCTCCCATCCGCTTTCTGGCCA

TATCTTTTAGTCTGCGTCTGCGTTTGTATCTAAATCTATGGGTTGTTTCT

GCATCAGTATCTGTATCTGTGTCTC

CGCGTTTTTCTCGCCGTTGTCTGCCTTACAAAACGTGTTCATTACAAGAA

TAGGTAACGGTAGGAGTAGTACTAATAGTAATAGTAATAGTAATCCGATC

GGCCAAATGTGTCAGAGCTGAAACGTCATGATTAATGATGCAAACATGTG

GGCCCGTCTCCGTCTGCCTCTCTGTGTCCGACCGACTCCGACTACGATTC

CCAATCCGACTGCGAGTCCGGCTACCTTTACGACTCGAATGCGTTCAGGG

GGCAGAGACATAAACAGCCCGGGGACAGGGCACGGCATGGGAATGGGAAT

GGGAATGGAATTGGGAATGCGAACGGTAACGTTTTATCAAGGTAAATTAG

AAGACGAGACACCCAAAATGTAAAC

ATCGAACTGGGTAGCTCTTGGACATTAGGCGAAATTAGAAATTAATCCTG

CCACTAAACCACAGATGGAAACACTTCCAAGGGGGATGGTGTGGGAAGAT

TCGACTCCGATGGTCTGAGCAGGGGTAGAGTATCAATTTTTTGTCTACTT

CTTCTTTTGGGGGGCGGAACTTTAGAGGTCAGGCAAAATTAATGGGAAAT

CCTCATGACTGACAAGTCGGAGTAATAGCATATACACACATCATAGTTGA

TCTATCGAGTGAAGCTCATCGAGGAAGTTCCAAATTATATTTCGAGAAAA

AAGGCCTTATATTGGGGAATTGATAAAATAGATCATATATTTATAACCCT

TATCTAAGAAGGCTACGTATTTGAAAGGTTCATTCAGCATTACCG

>EO053_FG3m

CCGGAGCGGCACAATTAGTCTTGCCCATAACAGCAGCTAATAGTTTCGTA

AATAGTTTTGGCCAATATTTACATGAGTTGCCTTCTGCCTGGGCTGCTTG

TGGGTTTTGTTCCTTTTGTCTTTTGCTGCCATTGTGAAAATATTTGTCTT

CAAACGACCCATTGTCCCTGCCCGCTCTCCTGTCATTCGGCACGCATCGG

CTACACTCGGAAATATTTACCCCAATTGTATTTCAACTTTCTTTTCACTT

ATAAAATATTTCTGCAAAATATTCTGAGTTTTAAGTTTGTTAAATAGATT

AAAATGACTTGGTTTTGGAAATAAATATTGTAGAACGCGTTCAGATAAAA

GCCGTCCTTTTCAGGTAAGGTAGTAACCAAGAAGTGGCAAGGTAAATACA

ATATTCTGCTACATACTGAAATTTTTTCGACAACAATTTATGTATCTAAA

TAAAAAATATCTCTGAGTGCACACGCATCCGCAGTCGTCGCCGTTCACTG

CTGACGTGCGTCTGTCTCAAGCTCGTCGTCTCCCATCCGCTTTCTGGCCA

TATCTTTTAGTCTGCGTCTGCGTTTGTATCTAAATCTATGGGTTGTTTCT

GCATCAGTATCTGTATCTGTGTCTC

CGCGTTTTTCTCGCCGTTGTCTGCCTTACAAAACGTGTTCATTACAATCC

GCTTGCCATTGCTTCTGCTGCAGCCGCTGCCGCTGCCGCTGCCGAATCGA

TTAACCCGTGTGACTCTAGTCCCATGACGTCGGCCGTCGTAAAACATGTG

GGCCCGTCTCCGTCTGCCTCTCTGTGTCCGACCGACTCCGACTACGATTC

CCAATCCGACTGCGAGTCCGGCTACCTTTACGACTCGAATGCGTTCAGGG

GGCAGAGACATAAACAGCCCGGGGACAGGGCACGGCATGGGAATGGGAAT

GGGAATGGAATTGGGAATGCGAACGGTAACGTTTTATCAAGGTAAATTAG

AAGACGAGACACCCAAAATGTAAAC

ATCGAACTGGGTAGCTCTTGGACATTAGGCGAAATTAGAAATTAATCCTG

CCACTAAACCACAGATGGAAACACTTCCAAGGGGGATGGTGTGGGAAGAT

TCGACTCCGATGGTCTGAGCAGGGGTAGAGTATCAATTTTTTGTCTACTT

CTTCTTTTGGGGGGCGGAACTTTAGAGGTCAGGCAAAATTAATGGGAAAT

CCTCATGACTGACAAGTCGGAGTAATAGCATATACACACATCATAGTTGA

TCTATCGAGTGAAGCTCATCGAGGAAGTTCCAAATTATATTTCGAGAAAA

AAGGCCTTATATTGGGGAATTGATAAAATAGATCATATATTTATAACCCT

TATCTAAGAAGGCTACGTATTTGAAAGGTTCATTCAGCATTACCG

>EO053_FG4m

CCGGAGCGGCACAATTAGTCTTGCCCATAACAGCAGCTAATAGTTTCGTA

AATAGTTTTGGCCAATATTTACATGAGTTGCCTTCTGCCTGGGCTGCTTG

TGGGTTTTGTTCCTTTTGTCTTTTGCTGCCATTGTGAAAATATTTGTCTT

CAAACGACCCATTGTCCCTGCCCGCTCTCCTGTCATTCGGCACGCATCGG

CTACACTCGGAAATATTTACCCCAATTGTATTTCAACTTTCTTTTCACTT

ATAAAATATTTCTGCAAAATATTCTGAGTTTTAAGTTTGTTAAATAGATT

AAAATGACTTGGTTTTGGAAATAAATATTGTAGAACGCGTTCAGATAAAA

GCCGTCCTTTTCAGGTAAGGTAGTAACCAAGAAGTGGCAAGGTAAATACA

ATATTCTGCTACATACTGAAATTTTTTCGACAACAATTTATGTATCTAAA

TAAAAAATATCTCTGAGTGCACACGCATCCGCAGTCGTCGCCGTTCACTG

CTGACGTGCGTCTGTCTCAAGCTCGTCGTCTCCCATCCGCTTTCTGGCCA

TATCTTTTAGTCTGCGTCTGCGTTTGTATCTAAATCTATGGGTTGTTTCT

GCATCAGTATCTGTATCTGTGTCTC

CGCGTTTTTCTCGCCGTTGTCTGCCTTACAAAACGTGTTCATTACAATCC

GCTTGCCATTGCTTCTGCTGCAGCCGCTGCCGCTGCCGCTGCCGCCGATC

GGCCAAATGTGTCAGAGCTGAAACGTCATGATTAATGATGCCCCACGTGT

TTAAATGAGAATGAGTAAGAGAGTGTGAATCAATCAGACGACTACGATTC

CCAATCCGACTGCGAGTCCGGCTACCTTTACGACTCGAATGCGTTCAGGG

GGCAGAGACATAAACAGCCCGGGGACAGGGCACGGCATGGGAATGGGAAT

GGGAATGGAATTGGGAATGCGAACGGTAACGTTTTATCAAGGTAAATTAG

AAGACGAGACACCCAAAATGTAAAC

ATCGAACTGGGTAGCTCTTGGACATTAGGCGAAATTAGAAATTAATCCTG

CCACTAAACCACAGATGGAAACACTTCCAAGGGGGATGGTGTGGGAAGAT

TCGACTCCGATGGTCTGAGCAGGGGTAGAGTATCAATTTTTTGTCTACTT

CTTCTTTTGGGGGGCGGAACTTTAGAGGTCAGGCAAAATTAATGGGAAAT

CCTCATGACTGACAAGTCGGAGTAATAGCATATACACACATCATAGTTGA

TCTATCGAGTGAAGCTCATCGAGGAAGTTCCAAATTATATTTCGAGAAAA

AAGGCCTTATATTGGGGAATTGATAAAATAGATCATATATTTATAACCCT

TATCTAAGAAGGCTACGTATTTGAAAGGTTCATTCAGCATTACCG

>EO053_FG5m

CCGGAGCGGCACAATTAGTCTTGCCCATAACAGCAGCTAATAGTTTCGTA

AATAGTTTTGGCCAATATTTACATGAGTTGCCTTCTGCCTGGGCTGCTTG

TGGGTTTTGTTCCTTTTGTCTTTTGCTGCCATTGTGAAAATATTTGTCTT

CAAACGACCCATTGTCCCTGCCCGCTCTCCTGTCATTCGGCACGCATCGG

CTACACTCGGAAATATTTACCCCAATTGTATTTCAACTTTCTTTTCACTT

ATAAAATATTTCTGCAAAATATTCTGAGTTTTAAGTTTGTTAAATAGATT

AAAATGACTTGGTTTTGGAAATAAATATTGTAGAACGCGTTCAGATAAAA

GCCGTCCTTTTCAGGTAAGGTAGTAACCAAGAAGTGGCAAGGTAAATACA

ATATTCTGCTACATACTGAAATTTTTTCGACAACAATTTATGTATCTAAA

TAAAAAATATCTCTGAGTGCACACGCATCCGCAGTCGTCGCCGTTCACTG

CTGACGTGCGTCTGTCTCAAGCTCGTCGTCTCCCATCCGCTTTCTGGCCA

TATCTTTTAGTCTGCGTCTGCGTTTGTATCTAAATCTATGGGTTGTTTCT

GCATCAGTATCTGTATCTGTGTCTC

CGCGTTTTTCTCGCCGTTGTCTGCCTTACAAAACGTGTTCATTACAATCC

GCTTGCCATTGCTTCTGCTGCAGCCGCTGCCGCTGCCGCTGCCGCCGATC

GGCCAAATGTGTCAGAGCTGAAACGTCATGATTAATGATGCAAACATGTG

GGCCCGTCTCCGTCTGCCTCTCTGTGTCCGACCGACTCATCAGCATCGGA

AACCGAATCAGTATCTGAATTAGCAAGGGCATCAGCGAATGCGTTCAGGG

GGCAGAGACATAAACAGCCCGGGGACAGGGCACGGCATGGGAATGGGAAT

GGGAATGGAATTGGGAATGCGAACGGTAACGTTTTATCAAGGTAAATTAG

AAGACGAGACACCCAAAATGTAAAC

ATCGAACTGGGTAGCTCTTGGACATTAGGCGAAATTAGAAATTAATCCTG

CCACTAAACCACAGATGGAAACACTTCCAAGGGGGATGGTGTGGGAAGAT

TCGACTCCGATGGTCTGAGCAGGGGTAGAGTATCAATTTTTTGTCTACTT

CTTCTTTTGGGGGGCGGAACTTTAGAGGTCAGGCAAAATTAATGGGAAAT

CCTCATGACTGACAAGTCGGAGTAATAGCATATACACACATCATAGTTGA

TCTATCGAGTGAAGCTCATCGAGGAAGTTCCAAATTATATTTCGAGAAAA

AAGGCCTTATATTGGGGAATTGATAAAATAGATCATATATTTATAACCCT

TATCTAAGAAGGCTACGTATTTGAAAGGTTCATTCAGCATTACCG

>EO053_FG6m

CCGGAGCGGCACAATTAGTCTTGCCCATAACAGCAGCTAATAGTTTCGTA

AATAGTTTTGGCCAATATTTACATGAGTTGCCTTCTGCCTGGGCTGCTTG

TGGGTTTTGTTCCTTTTGTCTTTTGCTGCCATTGTGAAAATATTTGTCTT

CAAACGACCCATTGTCCCTGCCCGCTCTCCTGTCATTCGGCACGCATCGG

CTACACTCGGAAATATTTACCCCAATTGTATTTCAACTTTCTTTTCACTT

ATAAAATATTTCTGCAAAATATTCTGAGTTTTAAGTTTGTTAAATAGATT

AAAATGACTTGGTTTTGGAAATAAATATTGTAGAACGCGTTCAGATAAAA

GCCGTCCTTTTCAGGTAAGGTAGTAACCAAGAAGTGGCAAGGTAAATACA

ATATTCTGCTACATACTGAAATTTTTTCGACAACAATTTATGTATCTAAA

TAAAAAATATCTCTGAGTGCACACGCATCCGCAGTCGTCGCCGTTCACTG

CTGACGTGCGTCTGTCTCAAGCTCGTCGTCTCCCATCCGCTTTCTGGCCA

TATCTTTTAGTCTGCGTCTGCGTTTGTATCTAAATCTATGGGTTGTTTCT

GCATCAGTATCTGTATCTGTGTCTC

CGCGTTTTTCTCGCCGTTGTCTGCCTTACAAAACGTGTTCATTACAATCC

GCTTGCCATTGCTTCTGCTGCAGCCGCTGCCGCTGCCGCTGCCGCCGATC

GGCCAAATGTGTCAGAGCTGAAACGTCATGATTAATGATGCAAACATGTG

GGCCCGTCTCCGTCTGCCTCTCTGTGTCCGACCGACTCCGACTACGATTC

CCAATCCGACTGCGAGTCCGGCTACCTTTACGACTATCCGTATGGACTTT

TTACTCTCACGCCCACTAAATTTTCACTTTACCGGCATGGGAATGGGAAT

GGGAATGGAATTGGGAATGCGAACGGTAACGTTTTATCAAGGTAAATTAG

AAGACGAGACACCCAAAATGTAAAC

ATCGAACTGGGTAGCTCTTGGACATTAGGCGAAATTAGAAATTAATCCTG

CCACTAAACCACAGATGGAAACACTTCCAAGGGGGATGGTGTGGGAAGAT

TCGACTCCGATGGTCTGAGCAGGGGTAGAGTATCAATTTTTTGTCTACTT

CTTCTTTTGGGGGGCGGAACTTTAGAGGTCAGGCAAAATTAATGGGAAAT

CCTCATGACTGACAAGTCGGAGTAATAGCATATACACACATCATAGTTGA

TCTATCGAGTGAAGCTCATCGAGGAAGTTCCAAATTATATTTCGAGAAAA

AAGGCCTTATATTGGGGAATTGATAAAATAGATCATATATTTATAACCCT

TATCTAAGAAGGCTACGTATTTGAAAGGTTCATTCAGCATTACCG

>EO053_FG7m

CCGGAGCGGCACAATTAGTCTTGCCCATAACAGCAGCTAATAGTTTCGTA

AATAGTTTTGGCCAATATTTACATGAGTTGCCTTCTGCCTGGGCTGCTTG

TGGGTTTTGTTCCTTTTGTCTTTTGCTGCCATTGTGAAAATATTTGTCTT

CAAACGACCCATTGTCCCTGCCCGCTCTCCTGTCATTCGGCACGCATCGG

CTACACTCGGAAATATTTACCCCAATTGTATTTCAACTTTCTTTTCACTT

ATAAAATATTTCTGCAAAATATTCTGAGTTTTAAGTTTGTTAAATAGATT

AAAATGACTTGGTTTTGGAAATAAATATTGTAGAACGCGTTCAGATAAAA

GCCGTCCTTTTCAGGTAAGGTAGTAACCAAGAAGTGGCAAGGTAAATACA

ATATTCTGCTACATACTGAAATTTTTTCGACAACAATTTATGTATCTAAA

TAAAAAATATCTCTGAGTGCACACGCATCCGCAGTCGTCGCCGTTCACTG

CTGACGTGCGTCTGTCTCAAGCTCGTCGTCTCCCATCCGCTTTCTGGCCA

TATCTTTTAGTCTGCGTCTGCGTTTGTATCTAAATCTATGGGTTGTTTCT

GCATCAGTATCTGTATCTGTGTCTC

CGCGTTTTTCTCGCCGTTGTCTGCCTTACAAAACGTGTTCATTACAATCC

GCTTGCCATTGCTTCTGCTGCAGCCGCTGCCGCTGCCGCTGCCGCCGATC

GGCCAAATGTGTCAGAGCTGAAACGTCATGATTAATGATGCAAACATGTG

GGCCCGTCTCCGTCTGCCTCTCTGTGTCCGACCGACTCCGACTACGATTC

CCAATCCGACTGCGAGTCCGGCTACCTTTACGACTCGAATGCGTTCAGGG

GGCAGAGACATAAACAGCCCGGGGACAGGGCAATTACGTTTCCGTTTCCG

TTTCCGTTCCGGTTTCCGTATCCATTGCCCGTTTTATCAAGGTAAATTAG

AAGACGAGACACCCAAAATGTAAAC

ATCGAACTGGGTAGCTCTTGGACATTAGGCGAAATTAGAAATTAATCCTG

CCACTAAACCACAGATGGAAACACTTCCAAGGGGGATGGTGTGGGAAGAT

TCGACTCCGATGGTCTGAGCAGGGGTAGAGTATCAATTTTTTGTCTACTT

CTTCTTTTGGGGGGCGGAACTTTAGAGGTCAGGCAAAATTAATGGGAAAT

CCTCATGACTGACAAGTCGGAGTAATAGCATATACACACATCATAGTTGA

TCTATCGAGTGAAGCTCATCGAGGAAGTTCCAAATTATATTTCGAGAAAA

AAGGCCTTATATTGGGGAATTGATAAAATAGATCATATATTTATAACCCT

TATCTAAGAAGGCTACGTATTTGAAAGGTTCATTCAGCATTACCG

>EO053_FG8m

CCGGAGCGGCACAATTAGTCTTGCCCATAACAGCAGCTAATAGTTTCGTA

AATAGTTTTGGCCAATATTTACATGAGTTGCCTTCTGCCTGGGCTGCTTG

TGGGTTTTGTTCCTTTTGTCTTTTGCTGCCATTGTGAAAATATTTGTCTT

CAAACGACCCATTGTCCCTGCCCGCTCTCCTGTCATTCGGCACGCATCGG

CTACACTCGGAAATATTTACCCCAATTGTATTTCAACTTTCTTTTCACTT

ATAAAATATTTCTGCAAAATATTCTGAGTTTTAAGTTTGTTAAATAGATT

AAAATGACTTGGTTTTGGAAATAAATATTGTAGAACGCGTTCAGATAAAA

GCCGTCCTTTTCAGGTAAGGTAGTAACCAAGAAGTGGCAAGGTAAATACA

ATATTCTGCTACATACTGAAATTTTTTCGACAACAATTTATGTATCTAAA

TAAAAAATATCTCTGAGTGCACACGCATCCGCAGTCGTCGCCGTTCACTG

CTGACGTGCGTCTGTCTCAAGCTCGTCGTCTCCCATCCGCTTTCTGGCCA

TATCTTTTAGTCTGCGTCTGCGTTTGTATCTAAATCTATGGGTTGTTTCT

GCATCAGTATCTGTATCTGTGTCTC

CGCGTTTTTCTCGCCGTTGTCTGCCTTACAAAACGTGTTCATTACAATCC

GCTTGCCATTGCTTCTGCTGCAGCCGCTGCCGCTGCCGCTGCCGCCGATC

GGCCAAATGTGTCAGAGCTGAAACGTCATGATTAATGATGCAAACATGTG

GGCCCGTCTCCGTCTGCCTCTCTGTGTCCGACCGACTCCGACTACGATTC

CCAATCCGACTGCGAGTCCGGCTACCTTTACGACTCGAATGCGTTCAGGG

GGCAGAGACATAAACAGCCCGGGGACAGGGCACGGCATGGGAATGGGAAT

GGGAATGGAATTGGGAATGCGAACGGTAAATGGGGCGACCTTGCCCGGCT

CCTCATCTCACAAACCCCGTGCCCA

ATCGAACTGGGTAGCTCTTGGACATTAGGCGAAATTAGAAATTAATCCTG

CCACTAAACCACAGATGGAAACACTTCCAAGGGGGATGGTGTGGGAAGAT

TCGACTCCGATGGTCTGAGCAGGGGTAGAGTATCAATTTTTTGTCTACTT

CTTCTTTTGGGGGGCGGAACTTTAGAGGTCAGGCAAAATTAATGGGAAAT

CCTCATGACTGACAAGTCGGAGTAATAGCATATACACACATCATAGTTGA

TCTATCGAGTGAAGCTCATCGAGGAAGTTCCAAATTATATTTCGAGAAAA

AAGGCCTTATATTGGGGAATTGATAAAATAGATCATATATTTATAACCCT

TATCTAAGAAGGCTACGTATTTGAAAGGTTCATTCAGCATTACCG

>EO053_TTAAm

CCGGAGCGGCACAATTAGTCTTGCCCATAACAGCAGCTAATAGTTTCGTA

AATAGTTTTGGCCAATATTTACATGAGTTGCCTTCTGCCTGGGCTGCTTG

TGGGTTTTGTTCCTTTTGTCTTTTGCTGCCATTGTGAAAATATTTGTCTT

CAAACGACCCATTGTCCCTGCCCGCTCTCCTGTCATTCGGCACGCATCGG

CTACACTCGGAAATATTTACCCCAATTGTATTTCAACTTTCTTTTCACTT

ATAAAATATTTCTGCAAAATATTCTGAGTTTTAAGTTTGTTAAATAGATT

AAAATGACTTGGTTTTGGAAATAAATATTGTAGAACGCGTTCAGATAAAA

GCCGTCCTTTTCAGGTAAGGTAGTAACCAAGAAGTGGCAAGGTAAATACA

ATATTCTGCTACATACTGAAATTTTTTCGACAACAATTTATGTATCTAAA

TAAAAAATATCTCTGAGTGCACACGCATCCGCAGTCGTCGCCGTTCACTG

CTGACGTGCGTCTGTCTCAAGCTCGTCGTCTCCCATCCGCTTTCTGGCCA

TATCTTTTAGTCTGCGTCTGCGTTTGTATCTAAATCTATGGGTTGTTTCT

GCATCAGTATCTGTATCTGTGTCTCCGCGTTTTTCTCGCCGTTGTCTGCC

TTACAAAACGTGTTCATTACAATCCGCTTGCCATTGCTTCTGCTGCAGCC

GCTGCCGCTGCCGCTGCCGCCGATCGGCCAAATGTGTCAGAGCTGAAACG

TCATGAggccTGATGCAAACATGTGGGCCCGTCTCCGTCTGCCTCTCTGT

GTCCGACCGACTCCGACTACGATTCCCAATCCGACTGCGAGTCCGGCTAC

CTTTACGACTCGAATGCGTTCAGGGGGCAGAGACATAAACAGCCCGGGGA

CAGGGCACGGCATGGGAATGGGAATGGGAATGGAATTGGGAATGCGAACG

GTAACGTTTTATCAAGGTAAATTAGAAGACGAGACACCCAAAATGTAAAC

ATCGAACTGGGTAGCTCTTGGACATTAGGCGAAATTAGAAATTAATCCTG

CCACTAAACCACAGATGGAAACACTTCCAAGGGGGATGGTGTGGGAAGAT

TCGACTCCGATGGTCTGAGCAGGGGTAGAGTATCAATTTTTTGTCTACTT

CTTCTTTTGGGGGGCGGAACTTTAGAGGTCAGGCAAAATTAATGGGAAAT

CCTCATGACTGACAAGTCGGAGTAATAGCATATACACACATCATAGTTGA

TCTATCGAGTGAAGCTCATCGAGGAAGTTCCAAATTATATTTCGAGAAAA

AAGGCCTTATATTGGGGAATTGATAAAATAGATCATATATTTATAACCCT

TATCTAAGAAGGCTACGTATTTGAAAGGTTCATTCAGCATTACCG

>zen2US

TGATGTGATGACACCAATTTATCTGAGCTCACTAAGGAGCAGAGGGGCCT

GCCGTTGTGACCACAAAATTGTTCGACCAAATGGCTTGCAAGTTGTCTTC

ACATCTTAACCATGCATAAGAGCTCTGACTAAATTATAGTAATGCTGGCT

TGCAGTCTCGTCTTGAATTGTGTATATAAAATCATAAAATTTAAGGTAAA

ATCTACAATTTGCTCTTTCTGTAAAGTCCTCCACTTAGATTGTACCAAAA

TGGAGAATGTAGAATCTTACGGATCATCTGATAGGCTACTCGGGGCTACA

ATCTCGTCTGAGAGATTTGTTTTTGGGTGGACTTATGCTATTCCCTCCCA

GAAAACCTAGTTTTAGTTTACATAAGCCCGGGCGATCGTAAATCTGTGCT

ATATAGCACGTAGCTCGGGGGATATGACCGATTTTATAGCGGGCGATTGG

ATTAGCTTGAAGTGACACTGTTTTTACAATCAGGGATCAGGGTCGGATCG

CTTAGCAATGCTAAGCAGGTAGCTCAACCCTTAGATATTCATAACTGACG

CGGAAAACCCCGGGAAAAACCAAGACCTTTCCCGTTCATGGCCAAAACAT

ACCTGGCCGGGGGATTGGGGTCTCGAAGGCATGAAAAAAGCTGGGCAGAT

CCGTAGGTATATAGGTAGGTAGGACTATCAGTGGATATTTTGGATGACAA

AGTGAACTTAGAACACACTTATTTTCAAATGATAGTCAGTTTTTATTTGG

ATAAGAATGGAAATATTTCTCAATACTGAAATATTATTTATCTATAAGCT

ATCTCGTAGGCCTAAAGTTGATTTAAAATTAAAATAATTTTTATCTAAGA

GTTAACGTTTAGGTGCCTTGAAAATGGAGATGTATTTTAATGTTTTCATC

ACAAAATGTTTCAGATCTTAAAATATATTACATTTGATTTTTTGCAAGTA

TTTATTTGACGAACTAAATAATATCATATAAATAAAACAAATAGATCCAG

ACGTAACATTAATATCCCCTGAATAATATTTCTCCGTGTGACGCTACTTA

GAAGTACGTCGGTTTCTCTGGAGCCTGGAACTCACCTTGATTCGGAGCCA

GGCTCAAAAGGACGCGACATCTACCAGCCCAGACCAGCCGTCTGGGAATT

TGAAAGTGTCGCAGGCGGGACAGGTAGGAACCAATCCGATCCGTCACGAA

GGGCTTCTGGGGTATATAA

>pbM2:20deletion_5’breakpoint

GTAGAACGCGTTCAGATAAAAGCC

>pbM2:20deletion_3’breakpoint

AGCTTCCAGAGTGGGAGTTTGGCTTG

>pbΔ2.1 HDR template fragment (homology arms joined by mixed XhoI/SalI site)

ACCGAAATTCGAGACAAACTCAAA

GGATTTTGGTTAAACGTTGTACGATAATTTCTCGGGCCCTT

TTCCCTTTAAAAATAATGACAAATCAGTTTGCAGATAAAAC

CGCAAAAAAAAGCTACCCAAAAATGTGGCAGAAAATAATAA

TCATAAAATTTACTTGGGCCAGCCGCCGGCTCAGGCTTAAC

TAATTGTAACAGATTTTTTTCTGAGTCATGTTGGAAAGGGA

AAGGCCAAGAAAAGCGAAAGAAAAATATGGAAAAACCCTCT

GGGGCGTAAACGTCTGGCCGGCATTATCCTGGCTGCGGTGG

GCCAATTAGAACCCTTTGTTTGTTCTGGCCAAGACGCCGTG

CACTTATCTCCATAATAAAGTCGTAACAATTAATTGAGCGA

GCCGAAAACGGGCCTCCTTACCCAAAAGATTGCAGTCCTTG

AGCCTTCCTGGAGCCTTTTCGTTGGCCCTGAAAGTTTTTCA

TCGTTTGACGCGTAATTAGTGCTCAAAAATTATTTGACGAC

CGCCAATTAATTTAATGCTCGGCGACATGATTTTACCTTTT

AGAGACGGTTATCCACGGCAGCTTAAGTTCTCTCGTTAAAT

GGACTTTAATTCTTAATTAAGCATCGGATTGTTGATAGGCT

CCATTAAAATCACCAATTTTTGTTGCAAAACATATATTTAC

TCTTTGTGGAATTTAATAATTTTTTATCACATCTTTTTCCT

GTGAATATGAGCTTGTTAATGTCAACCACATAATCGTGGGA

TATCAGTATCAGAGTGCCATGAAAACATTTCATCCCTCTCA

ATATGAGATTTTTCAAAAATCTCTTTGATTTTCTTAGAGCA

TTCTGAATGAAGGAATATCGCTGAATATTTATCGCTCCCCA

GCTTTTTTAGCATTCGCGCCGAATTCGTTTTGAATGAATTC

AACTGTAAAGGTTAAGGTTAAAGGTTAAGGTTAAAATTATA

AATGTGCGAATGACTCATCATAAATAAAATTAATAAAAGGC

ACCGCGAACCTGGCCCTGTCCTCTGCTTTTTTCTGACCCTG

GCGGACTTTGAGACCTTCATTGAGAAAAAGTACATCGCATC

AACATAAATATTATGATAAATGGTTTGTTATAATCTTAATC

CTCGGGGGGTAAGTTTTGAGTCGTGCCGCACGCATTTTTTA

TGCTCTTCCGCCCTTCCACCCACGAAACCATCCAGGACGAG

GATGAACTGCTTCTCCTCTCAATGGAGCAACATATGGTCGC

AGGGTGGATAATACAGATGGCGCTTCGAATGGATTGCCGCT

TCTCAATAGCCAGGGCCAACGAAGTCAAATTGTTTTTTATT

GGTTTACCCCCAACGCCGCTCCTTTAAGTGAAAGTATCGCA

TTGTCCTGGGGAGATCCGTTCGCGTATCTCGCAGATACTTG

GGAGGACGCGACATGTTGCTCGACAGTTACGAACTTTTCTA

GCAGTTCATTACGCCTAATTAACACTTGATACTGAACGGAA

GTTTTATTGCTTCCCCTTCATGTTCATGTAAACAGGCTAAC

AAAACAATTTCTGAGGCGAAATACCCATCTTTCAACTCTTG

CAACCCATTCTGCGATGTGACTTCAGCCGCAATACGCCGCA

CACATTAAAATTCAGGATGCGTTGCGAACAATTTGCGGTAA

ACAATGTGAGATTGCAAAGGGGGTGGGGTACGCGGATTCAA

ATTGTTATTTCAGCCAGGAAACGGAATTTACAAAATGCTGC

ATTTAATGATATGGCTCAACAGGCCCTTTTTGTTTGGAGTA

TCTTGAAGTTATTGCATGCTAAACAGGAAGAAAAATAAAAA

GGAACCATAACCCTAGTAATGGATAGAGGAGATTTTACTTC

TTCCAATCGCCTTTTCCAATAGTTACAATCGCGATCTTTTG

GCAACGAATTAACCGTTTCCCATTCACAGATCCGCCCACCT

GAGAATCATTTAGATCGATTACCACTTGACAAGGAATTAAT

TTGACAACGTTAACAAATTAAACTGTTAAACCAACACAAAC

ACACTAGTCCGCTCGCACACTGAGATAAACAAATGGTCGAC

GTCGAG

CTGATTGTTGTTGTTGCTGCTCTTGCGGGATGTCTTCT

TCTCCTTCATCCAGGGATACTCCGGCACCGAGTCCATTCCA

TCCGGCGTTTGAGGGACAACGCCAACCGGATATCCGACTCC

GACGCCGACATTGACATTAACGTTGACGCCTACTCCGCCAA

TGGCGCCGGATCCAACAGGTGTTCCGATTTTGGGACTCTCC

GGACTCAAGTGGTTGAGGAATTCAGCCATCGACGGCTGCGA

ATTGATGAACCCGCCCTCGGATGCTGTCATCCAATAGGCCG

TGTCCGCGCTCCGTTTATCACAGTTCGGGGTCATCTTGTTG

ACCATGAGCACCGACGGCACAGGTGGCACCCCCTGCTGTCC

GATACCAGGCTGACCAACACTCACTCCCACGCCACCCACAC

CACCCACGACGCCCGCACCACCGCAACCACCCACTGGCAGG

GACGTCTGTCCAGTTTGCACCTGCAGCGGGTTGAGTGGAGA

TTCCGATTTGATTTGGGTGCCCATTGAGGTCGTGTCCAAGG

AGCTGCAGACTTCTTGCATAGCTTGGGTATAATTTTTTTTA

GATTCTTTCTATTTCAATTCCTGAGCGTATTAGATGTTCCT

CAGCTTGGGAACCTGGAAAGGAAAGCCATTTTTGTAATCTC

ATAATAATGTTATCACGATTCAGTTCCCAGGAAGTTAGAGG

TTTCACTGTTCTTATCGAATGTGAAATTGAATAATGTATGT

ATATACTTCATTAAGATATGTTATCATTATCTTCATTAACC

TTTTAAGAAACAAAGACATCGTCACTTTACTATGCTATTCA

ATGTTTATTTTATTATTATATTTATAATTTTTGAATTTTTA

TTTATATATTTTGAATATATATAATTTCAATATAATGTACG

TATATACTTCATTAAGATATGTCATTATCTTCATTAACATT

TTAAGAAACAAAAACATCGTCACTTTACTATGCTAATCAAT

GTTTATTTTATTATTACCGATCCAATTTTTCACATTGCAAC

TGTCAAAAACTTGACTATTTTTAAATTACATATTCCAATTT

ATATTTCATTAGTCTCTCAGTTTTCTGTGCCAGAAATTCAT

TTGTAGTCTGCATTTTTACTATTGCTCTTATAGAAATTGTT

AGGCCTTCACTTTAAATTTATGGCCACCTAATTTCGATTGA

GATTTGACAATATGTATTGTATGTATGTACAATACATACAA

CCTGTCCTACAATGTTACCTAAATTGGCTTGGTTCGCATAC

ATTATTTTACATTATTTTTTTAAGGATTAATAGTCAGTAAA

TAAATTAACATATTTTAAACAGAAAAAGTTATAAAGCTGTT

ACATATCCTCATCTAAGAAATGCCGCATAGCTAATTTCCTA

GGAAGTATAAATGAATAAGAAAATTCGAATATATTTTCCGA

TTCCGCCAATTAAAGATTTGAAATATTTCTTGCAGTAAACA

TTTTGCCGACTTAAAAGGCTCGTATTTTAGACTAACATGTT

GTATCAAAAACGAAATAACGACACGCTTTGATTTTCTCGTG

CTTTAAAAGGATACACTTATTTTTAAATGTAGGTAAGAAAT

ATGTGTAAGATAATTTCGTACCATATTAAGTTGATCATTTA

CAGACCACAGTATTCCATATTTCTCCTCTTACGTTGACATA

TGCGTTCTTTGTGGAAATTTCACCGGTATCACTTGGGTCAC

CGTTTTCACTATTGTCTGCACTCGCTCATAGTAACTGGCTT

AACTTTTTGTTTGTTTTTTTTTTTTAGTTTCAACCGGCACA

ACTTTTGCGATGCGGGTTTTGCGTAAAACTTCAGTGTGTCA

GGGCTTATCTGTGGGC
